# Production of riboflavin and related cofactors by biotechnological processes

**DOI:** 10.1186/s12934-020-01302-7

**Published:** 2020-02-13

**Authors:** Shuang Liu, Wenya Hu, Zhiwen Wang, Tao Chen

**Affiliations:** Frontier Science Center for Synthetic Biology and Key Laboratory of Systems Bioengineering (Ministry of Education), SynBio Research Platform, Collaborative Innovation Center of Chemical Science and Engineering (Tianjin), School of Chemical Engineering and Technology, Tianjin University, Tianjin, 300072 People’s Republic of China

**Keywords:** Riboflavin, Flavin cofactor, Biotechnology

## Abstract

Riboflavin (RF) and its active forms, the cofactors flavin mononucleotide (FMN) and flavin adenine dinucleotide (FAD), have been extensively used in the food, feed and pharmaceutical industries. Modern commercial production of riboflavin is based on microbial fermentation, but the established genetically engineered production strains are facing new challenges due to safety concerns in the food and feed additives industry. High yields of flavin mononucleotide and flavin adenine dinucleotide have been obtained using whole-cell biocatalysis processes. However, the necessity of adding expensive precursors results in high production costs. Consequently, developing microbial cell factories that are capable of efficiently producing flavin nucleotides at low cost is an increasingly attractive approach. The biotechnological processes for the production of RF and its cognate cofactors are reviewed in this article.

## Introduction

**Fig. 1:**
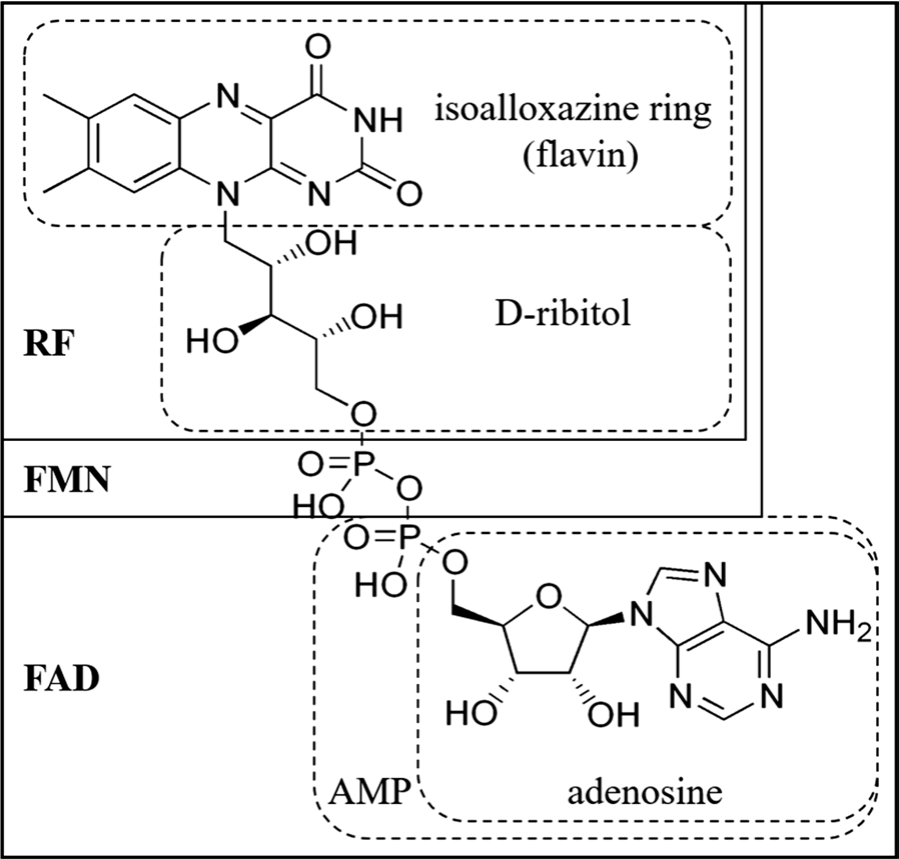
Chemical structure and nomenclature of flavins

Flavins are a set of pteridine-based yellow organic compounds derived from the isoalloxazine ring (Fig. 1). Riboflavin (RF, commonly known as vitamin B2) is the central source of all biologically important flavins. RF was discovered in 1879 as a yellow pigment from milk and its chemical structure was deciphered in the 1930s [1]. Previous reviews have reviewed exhaustively the discovery history of RF [1–3]. Its derivatives flavin mononucleotide (FMN) and flavin adenine dinucleotide (FAD) are indispensible as active groups in the majority of flavoproteins/flavo-coenzymes. These flavo-coenzymes play key roles in multiple crucial physiological functions, including redox homeostasis, protein folding, DNA repair, fatty acid β-oxidation, amino acid oxidation, and choline metabolism [4–8]. Flavins are broadly distributed in tissues, but they are rarely present as free RF. Instead, most are bound to flavoproteins, mainly as FAD and lesser amounts as FMN [9, 10]. Crystallographic studies revealed that the majority of flavin-protein interactions proceed via the N(10)-ribityl side [11]. While, the 5′-OH modification of the ribityl chain is generally used as a “handle” by proteins [12], pyrophosphate binding is also a significant component of molecular recognition in FAD containing proteins [13]. Humans and livestock must obtain RF from the diet because they have lost the ability of its de-novo synthesis.

Commercial RF is mainly used in the food, feed and pharmaceutical industries. Small-scale production of RF by probiotic lactic acid bacteria (e.g. *Lactobacillus plantarum*) has the potential for the development of dairy- and cereal-based functional foods for the in situ delivery of RF to consumers [14–20]. RF deficiency may lead to increased risk of cardiovascular disease, impairment of iron metabolism and night blindness. Flavins are used for the treatment of ariboflavinosis, a condition marked by lesions in the corners of the mouth, on the lips, and around the nose and eyes, or as general health supplements in the case of malnutrition [21]. High doses of RF promote the recovery of some motor functions in patients with Parkinson’s disease [22]. Supplementation with RF helps with the treatment of lactic acidosis [23]. Intravenous injection of Cardiocrome^®^, which contains FMN, is fairly effective for the management of patients with mitochondrial encephalomyopathy [24]. FAD is also considered potential treatment for diseases such as Friedreich ataxia [25] and chronic granulomatous disease [26]. As excellent redox coenzymes, FMN and FAD are important biochemical reagents with significant application potential in the enzyme industry.

Nowadays, the industrial production of RF is exclusively accomplished by microbial fermentation without the involvement of chemical synthesis. Chemical synthesis of RF essentially consisted of six to eight chemical steps starting from d-glucose or D-ribose. Please refer to earlier literature for more information about chemical methods of RF synthesis [2, 3]. The industrial strains used for RF production mainly are mainly derived from the bacterium *Bacillus subtilis* (*B. subtilis*) and the fungus *Ashbya gossypii* (*A. gossypii*) [27]. The yeast *Candida famata* (*C. famata*) was once also used for the industrial production of RF [28], but the ADM Company (USA) has stopped this industrial process because it was unprofitable due to the low stability of the strain [1, 29, 30]. As the engineered RF production strains are typically acquired by a combination of mutagenesis and genetic engineering, the genetic background is often complex and can be perplexing. For example, *B. subtilis* carries several antimicrobial resistance genes, and thus carries a risk of spreading multidrug resistance [31]. FMN and FAD are synthesized by a combination of biotechnological and chemical processes. The biosynthesized RF is converted into FMN using a non-specific phosphorylation reagent. Nevertheless, several biotechnological processes for the production of FMN and/or FAD production have been developed, including approaches based on whole-cell catalysis [32–34], the cofactor trapping method [35], and the fermentation of microorganisms modified by metabolic engineering [36–38]. However, the production of FMN and FAD by fermentation is rather low compared with the industrial production of RF. This review covers the recently developed biotechnological processes for the production of RF, as well as specifically FMN and FAD.

## Biosynthetic pathways of RF, FMN and FAD in bacteria and fungi

### Overview of the biosynthetic pathways of flavins

**Fig. 2:**
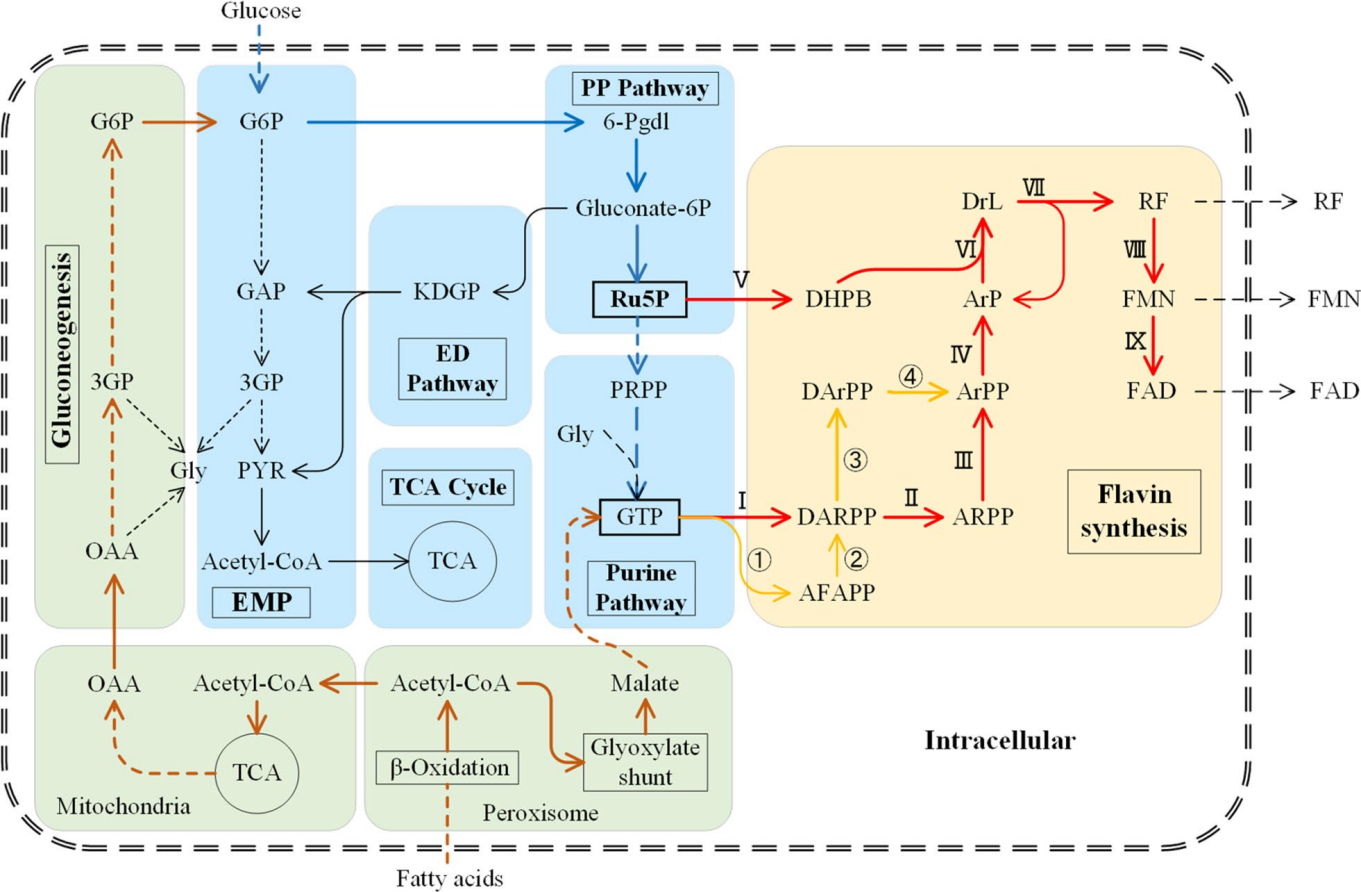
Schematic overview of the relevant pathways for flavin production. *(Pathways)* PP pathway, the pentose phosphate pathway; TCA, the tricarboxylic acid cycle; EMP, the Embden–Meyerhof pathway/glycolysis; ED, the Entner–Doudoroff pathway. *(Metabolites)* G6P, Glucose-6-phosphate; GAP, Glyceraldehyde 3-phosphate; 3GP, Glycerate 3-phosphate; PYR, Pyruvate; OAA, Oxaloacetate; 6-Pgdl, 6-Phospho-d-glucono-1,5-lactone; Gluconate-6P, 6-Phospho-d-gluconate; PRPP, Phosphoribosylpyrophosphate; Ru5P, Ribulose 5-phosphate; GTP, Guanosine 5′-triphosphate; Gly, Glycine; GTP, guanosine 5′-triphosphate; DHPB, L-3,4-Dihydroxybutan-2-one 4-phosphate; DARPP, 2,5-Diamino-6-(1-d-ribosylamino)pyrimidin-4(3H)-one 5′-phosphate; AFAPP, 2-Amino-5-formylamino-6-(5-phospho-d-ribosylamino)pyrimidin-4(3H)-one; DArPP, 2,5-Diamino-6-(1-d-ribitylamino)pyrimidin-4(3H)-one 5′-phosphate; ARPP, 5-Amino-6-(ribosylamino)-2,4-(1H,3H)-pyrimidinedione 5′-phosphate; ArPP, 5-Amino-2,6-dioxy-4-(5′-phospho-d-ribitylamino)pyrimidine; ArP, 5-Amino-6-(1-d-ribitylamino)uracil; DrL, 6,7-Dimethyl-8-(D-ribityl)lumazine; RF, Riboflavin; FMN, Flavin mononucleotide; FAD, Flavin adenine dinucleotide; *(Enzymes that catalyze the Reactions)* I, GTP cyclohydrolase II, (EC: 3.5.4.25); ①, GTP cyclohydrolase IIa, (EC: 3.5.4.29); ②, AFAPP deformylase (EC: 3.5.1.102); II & III, deaminase (EC: 3.5.4.26) & reductase (EC: 1.1.1.193); ③ & ④, reductase (EC: 1.1.1.302) & deaminase (EC: 5.4.99.28); IV, 5-amino-6-(5-phospho-d-ribitylamino)uracil phosphatase (EC: 3.1.3.104); V, DHPB synthase (EC: 4.1.99.12); VI, 6,7-dimethyl-8-ribityllumazine synthase (EC: 2.5.1.78); VII, RF synthase (EC: 2.5.1.9) VIII, RF kinase; IX, FMN adenylyltransferase/FAD synthetase

All plants and fungi, as well as most bacteria, are capable of producing RF [1]. The biosynthesis of flavins starts from GTP and Ru5P, and RF can be further converted into FMN and FAD (Fig. 2). Fungi such as the flavinogenic *A. gossypii* prefer oils as substrates, while the bacteria such as *B. subtilis* and *Escherichia* *coli* (*E. coli*) tend to utilize carbohydrates as carbon source. The biosynthetic pathways leading from the substrate to flavins encompass β-oxidation, the glyoxylate cycle, TCA cycle, gluconeogenesis, the PP pathway (oxidative branch), the purine pathway and the flavin synthesis pathway. The biotechnological engineering of flavin overproducing strains is always accomplished by overexpression of genes in the relevant pathways, suppression of competing pathways, and disruption of regulatory genes responsible for feedback inhibition.

### Biosynthesis of the precursors

As the precursor of RF, GTP serves as the donor of the pyrimidine ring and the nitrogen atoms of the pyrazine ring, as well as the ribityl side chain of the vitamin [39]. Ru5P is isomerized to ribose-5-phosphate (R5P) in the non-oxidative branch of the PP pathway. R5P is further phosphorylated to form PRPP, which in turn is converted into GTP via a series of enzyme-catalyzed reactions of the purine biosynthesis pathway. While similar, the biochemical pathways of GTP synthesis from Ru5P in bacteria and fungi show a different preference for carbon sources. Fungi such as *A. gossypii* prefer oils, which are cleaved into fatty acids and glycerol by the extracellular lipase. The fatty acids are further converted into acetyl-CoA via the β-oxidation pathway, which in turn is transformed into G6P through the glyoxylate cycle, TCA cycle and gluconeogenesis. The resulting G6P is used for the synthesis of Ru5P through the oxidative branch of the PP pathway. A shunt also converts a part of the acetyl-CoA into OAA, which is further transformed into glycine for purine synthesis. By contrast, bacteria such as *B. subtilis* and *E. coli* prefer glucose instead of oil, and can therefore directly synthesize G6P without activating gluconeogenesis. The further path of G6P via the PP pathway towards the synthesis of RF remains the same. Detailed reviews of the overall pathway of RF synthesis from carbon precursors can be found elsewhere [2, 27].

## Enzymes involved in the synthesis of RF, FMN and FAD

### Key enzymes for the synthesis of RF

A schematic of the RF synthesis pathway is shown in Fig. 2. RF biosynthesis starts from GTP (reaction I catalyzed by GTP cyclohydrolase II or ① + ② catalyzed by GTP cyclohydrolase III and formamide lyase). GTP is converted into DARPP as the first committed intermediate, which is further converted into ArPP by serial deamination of the pyrimidine ring and reduction of the ribosyl side chain (reactions II and III catalyzed by pyrimidine deaminase and reductase). This step also works in the reverse order (reactions ③ + ④). ArPP is then converted into ArP (reaction IV) by ArPP phosphatase. The penultimate reaction (reaction VI catalyzed by lumazine synthase) is the condensation of ArP and another intermediate (DHPB generated from Ru5P by DHPB synthase, reaction V), resulting in DrL. The final step in RF biosynthesis (reaction VII) involves dismutation of two molecules of DrL including an exchange of a 4-carbon unit, which results in a molecule of RF and the regeneration of a molecule of ArP. This step of the reaction is catalyzed by RF synthase. GTP cyclohydrolase II and DHPB synthase were identified as the rate-limiting enzymes for the biosynthesis of RF in an industrial *B. subtilis* strain [40]. A mutant of *B. subtilis ribA* encoding cyclohydrolase II showed a twofold increase in the activity of this enzyme [41]. Earlier publications offer detailed reviews of the biosynthesis pathway of RF and related enzymes [1, 2, 42, 43].

The mechanism of ArPP dephosphorylation was unclear until relatively recently. In 2013, researchers found that enzymes from the haloacid dehalogenase (HAD) superfamily catalyze the dephosphorylation of ArPP. At least two phosphatases (encoded by *yigB* and *ybjI,* respectively) belonging to the HAD family were found to dephosphorylate ArPP and FMN in *E. coli* [44]. Individual deletion of *ybjI* or *yigB* did not result in RF auxotrophy [45]. A study from 2011 showed that an FMN-specific hydrolase (AtcpFHy1) from the HAD superfamily was able to catalyze the dephosphorylation of FMN [46]. A few years later, in 2016, the plastidic FMN hydrolase AtcpFHy1 (*At1g79790*) together with the gene products of *At4g11570* and *At4g25840*, were identified as having ArPP phosphatase activity [47]. In 2015, the HAD superfamily phosphatase EFI-501083 from *Bacteroides thetaiotaomicron* (*B. thetaiotaomicron*) was predicted to catalyze the dephosphorylation of ArPP according to covalent docking models [48]. In the same year, another HAD enzyme encoded by the *ycsE* gene in *B. subtilis* was confirmed to catalyze the dephosphorylation of ArPP and FMN [49]. Just like in *E. coli*, the deletion of *ycsE* did not lead to RF auxotrophy. In summary, the dephosphorylation of ArPP is catalyzed by multiple enzymes from the HAD superfamily, most of which also possess FMN phosphatase activity. Moreover, the deletion of individual ArPP phosphatase genes does not lead to RF auxotrophy, indicating the existence of multiple redundant isoenzymes.

Generally, the overexpression of genes responsible for the synthesis of RF is an efficient method for enhancing RF production. In *B. subtilis*, flavinogenesis is encoded by the RF biosynthesis operon (RF operon or *rib operon*), encompassing five non-overlapping genes (*ribGBAHT*) and three regulatory elements (*ribO* regulatory region harboring the main promoter P1 and two additional internal promoters, P2 and P3). It contains all proteins required for RF biosynthesis except for ArPP phosphatase [50, 51]. Industrial RF producer strains of *B. subtilis* were successfully constructed by overexpressing the RF operon, even though it does not contain the phosphatase gene [40, 52]. In *A. gossypii,* overexpression of the *RIB* genes also significantly promoted the yield of RF [53]. In *C. famata*, overexpression of the genes *RIB1* and *RIB7*, respectively encoding GTP cyclohydrolase II and riboflavin synthase, led to increased production of RF [30]. In *E. coli,* overexpression of an artificial RF operon called EC10 (P*trc*-*ribABDEC*) was used to successfully engineer RF overproduction [54].

### Enzymes for the biosynthesis of FMN and FAD

FMN is produced via phosphorylation by RF kinase (RFK, EC 2.7.1.26), and is further transformed into FAD by FMN adenylyltransferase (FMNAT, EC 2.7.7.2). In most prokaryotes, the synthesis of FMN and FAD is executed by a bifunctional RFK/FMNAT enzyme, which is usually known as FAD synthetase (FADS). The C-terminal domain catalyzes FMN synthesis from RF (RFK activity), and the N-terminal domain transforms FMN into FAD (FMNAT) [55–61]. In addition to bifunctional FADS, bacteria such as *B. subtilis* [62] and *Streptococcus agalactiae* (*S. agalactiae*) [63] possess monofunctional enzymes with only RFK activity. While this is rare in prokaryotes, RFK and FMNAT activity are generally split into two different enzymes in most eukaryotes [64–66].

The monofunctional RFKs show sequence and structural homology with the C-terminal module of the bifunctional enzymes. However, eukaryotic FMNATs share little or no sequence similarity to the prokaryotic FMNAT-module, which makes it a potential antimicrobial target. Crystal structure of RFK and FADS enzymes from other species, such as *Thermotoga maritima* (*T. maritima*) [56, 57] and yeast [67, 68] were also solved. The bifunctional *Ca*FADS from *Corynebacterium ammoniagenes* (*C. ammoniagenes*) folds into a dimer-of-trimers assembly with a head-to-tail arrangement within each trimer [69–71]. The head-to-tail arrangement of *Ca*FADS causes the RFK and FMNAT catalytic sites of the two neighboring protomers to approach and influence the stabilization of assemblies, catalysis and ligand binding [61]. Moreover, it also leads to cross-talk between the RFK and FMNAT modules of neighboring protomers [72]. Research on energetically favored interactions of the FMNAT module of *Ca*FADS by molecular docking and molecular dynamics simulations revealed that the RFK module negatively influences FMN binding at the interacting FMNAT module, weakening its activity [73]. Binding of FMN and ADP ligands triggers dramatic structural changes in the RFK module [71].

Both monofunctional and bifunctional enzymes have been modified for improved activity by mutation of key residues. The D181A variant of the monofunctional yeast FMNAT was found to have a much faster turnover rate and attenuated product inhibition by FAD [68]. Point mutations in loop L1c-FlapI, loop L6c, and helix α1c of the RFK module of *Ca*FADS had a great effect on the steady-state kinetic parameters for both the RFK and the FMNAT activity [61]. Point mutations at R66 of *Ca*FADS mildly affected the ligand binding and kinetic properties of the FMNAT module but significantly impaired the RFK turnover. This was especially notable because R66 is located in the FMNAT module [72].

Expression of FADS in *C. ammoniagenes* was used to enable the production of FAD from FMN or RF, as well as for the production of FMN from RF [32, 34]. Overexpression of the FMN1 (encoding monofunctional RFK) and FAD1 (encoding monofunctional FADS) genes from *Debaryomyces* *hansenii* (*D. hansenii*) in the yeast *C. famata* led to the overproduction of FMN and FAD [36, 37]. In *E. coli*, the overproduction of FMN and FAD was also achieved by overexpressing the gene *ribF* gene encoding a bifunctional RFK/FMNAT [38].

## Microbial cell factories for the production of RF

Nowadays, commercial RF production is exclusively accomplished by microbial fermentation [2]. The industrial RF production strains are mostly derived from the bacterium *B. subtilis* and the flavinogenic fungus *A. gossypii*. Nevertheless, other species are also engineered for the production of RF, including *C. famata*, *C. ammoniagenes*, *E. coli*, *Pichia guilliermondii* (*P. guilliermondii*) and *Eremothecium gossypii* (*E. ashbyi*). The titer of RF in typical industrial processes is around 26–30 g/L [74, 75].

The industrial RF production strains were typically developed by a combination of mutagenesis and several rounds of genetic engineering. The mutagenesis process consists of several screening steps for resistance to different antimetabolites. Structural analogues of RF, roseoflavin (RoF^r^), different purine analogues such as 8-azaguanine (Az^r^), thioguanine and 8-azaxanthine, as well as decoyinine (Dc^r^) and the glutamine antagonist methionine sulfoxide [52, 76–78], have been successfully used for the screening of RF overproducing *B. subtilis* mutants. Although *A. gossypii* is not sensitive to the structural analogues of purines or RF, antimetabolites of itaconate and oxalate, as well as inhibitors of isocitrate lyase (ICL), were successfully applied [79–83]. The RF over-producer *A. gossypii* was also successfully isolated by analogous mutagenesis [84].

Metabolic engineering is the science of rewiring the metabolism of cells to enhance the production of native metabolites or to endow cells with the ability to produce new products [85]. Metabolic engineering involves the continuous improvement of cells through several rounds of genetic engineering [86]. Many RF overproducing strains have been constructed through metabolic engineering (Table 1). According to the metabolic pathway, the strategies of metabolic engineering for RF production can be classified into modifications of the RF synthetic pathway, the purine pathway, the central carbon metabolism, the synthesis of glycine, cell-scale optimization, and so on. In general, metabolic engineering for enhanced RF production was achieved by overexpressing the synthetic pathways of RF or its precursors, which was accomplished by the combination of direct gene duplication, replacement of the native promoter with a strong one, disruption of competing pathways, or modification of regulatory genes. Other strategies focused on the improvement of the host traits, for example by reducing the maintenance metabolism.

**Table 1:** Metabolic engineering for overproduction of RF by gene manipulation

Organism	GOI^a^ and Manipulation^b^	Riboflavin titers^c^	Improvement	References
Overexpression of the RF synthesis pathway				
*B. subtilis*	*ribA* +	^c−1^	25%	[40]
*B. subtilis*	*rib* operon +	0.4–0.7	Tenfold	[52]
*B. subtilis*	*rib* operon +	4.3	27%	[87]
*A. gossypii*	*RIB* genes +	0.327	3.1-fold	[53]
*E. coli*	*ribABDEC* +	0.229	–	[54]
*C. ammoniagenes*	*rib* genes +	15.3	16-fold	[88]
*C. famata*	*RIB1* +*, RIB7* +	16.4	62-fold	[30]
*E. ashbyi*	*RIB1* +*, RIB3* +	0.331	1.44-fold	[89]
*L. lactis*	*ribGBAH*	0.024	–	[90]
Overexpression of the purine biosynthesis pathway				
*B. subtilis*	*purF* +	~ 5.1	31%	[91]
*B. subtilis*	ΔpurR, purF*	0.827	Threefold	[92]
*A. gossypii*	*prs *+ *, AGR371C* +*, AGL080C* +	0.05	80%	[93]
*A. gossypii*	*AER117W*+	~ 0.12^c−2^	40%	[94]
*A. gossypii*	*ADE4 *+ *, SHM*+	24.28 mg/g^c−3^	12-fold	[95]
*A. gossypii*	AgADE4*+	0.228	Tenfold	[96]
*A. gossypii*	*ΔAgURA3*	7.5 mg/g^c−4^	6.5	[97]
*E. coli*	(*ndk, gmk, purA, purF and prs*) +	0.388	72%	[98]
*L. fermentum*	*ΔfolE*	3.49 mg/L	50%	[99]
Optimization of the central carbon metabolism				
*B. subtilis*	*zwf*+	~ 0.8	25%	[100]
*B. subtilis*	*zwf *+ , *gnd*+	15.7	39%	[101]
*B. subtilis*	*fbp *+ , *pckA *+ , *gapB*+	13.36	27.8%	[102]
*B. subtilis*	*ΔccpN*	~ 13	~ 28%	[103]
*E. coli*	*Δpgi, Δedd, Δeda*	0.56	–	[54]
Enhanced synthesis of glycine				
*A. gossypii*	*GLY1*+	~ 16 mg/g^c−4^	Ninefold	[104]
*A. gossypii*	*ΔSHM2*	9.6 mg/g^c−4^	Tenfold	[105]
*A. gossypii*	*AGX1*+	~ 0.15	30%	[106]
Other strategies				
*B. subtilis*	*ΔcydC*	12.3	38%	[107]
*B. subtilis*	HSPs+	~ 0.3–0.35	23–66%	[108]

^a^GOI represents the gene of interest. *ribA*, DHPB synthase; *RIB1*, GTP cyclohydrolase II; *RIB7*, RF synthase; *RIB3*, DHPB synthase; *purF*, PRPP amidotransferase; *purR*, purine repressor PurR; *AGR371C* and *AGL080C*, PRPP synthetases; *prs*, PRPP synthetase; *purF*, PRPP amidotransferase; *AER117W*, IMP dehydrogenase; *ADE4*, PRPP amidotransferase; *SHM1* and *SHM2*, serine hydroxymethyltransferase; *AgURA3*, orotidine-5′-phosphate decarboxylase; *zwf*, glucose-6-phosphate dehydrogenase; *gnd*, 6-phosphogluconate dehydrogenase; *fbp*, fructose-1,6-bisphosphatase; *pckA*, phosphoenolpyruvate carboxykinase; *gapB*, glyceraldehyde-3-phosphate dehydrogenase; *ccpN*, gluconeogenic repressor CcpN; *pgi*, glucose-6-phosphate isomerase; *edd*, phosphogluconate dehydratase; *eda*, multifunctional 2-keto-3-deoxygluconate 6-phosphate aldolase and 2-keto-4-hydroxyglutarate aldolase and oxaloacetate decarboxylase; *GLY1*, threonine aldolase; *SHM2*, serine hydroxymethyltransferase; *AGX1*, alanine-glyoxylate aminotransferase; *cydC,* cytochrome bd oxidase; HSPs, heat shock proteins; *folE*, GTP cyclohydrolase I

^b^“+”indicates gene over-expression; “−” indicates gene downregulation; “Δ” indicates gene knockout; “*” indicates gene mutation

^c^The maximum RF titer of the engineered strains. Unit: g/L unless otherwise specified; ^c−1^, Strain VB2XL1 produced up to 25% more RF as compared to its parent strain RB50::[pRF69]n::[pRF93]m Ade; ^c−2^, total (intracellular +extracellular) RF concentration; ^c−3^, mg/g of biomass; ^c−4^, mg/g mycelium

### Overexpression of the RF synthesis pathway

Overexpression of the RF synthesis pathway is a proven effective method for enhancing RF production, and has been achieved through multiple methods. Overexpression of genes responsible for RF synthesis or the RF operon is one of the most common methods to improve the synthesis of RF. In *B. subtilis*, GTP cyclohydrolase II and DHPB synthase are rate-limiting enzymes and the introduction of an additional copy of the *ribA* gene encoding GTP cyclohydrolase II and DHPB synthase into the genome resulted in a 25% increase of the RF yield [40]. Strains with multiple copies of the RF operon on an integrative vector produced approximately tenfold more RF [52]. The heterologous expression of the *rib* operon from *Bacillus cereus* (*B. cereus*) ATCC14579 in the chromosome of *B. subtilis* led to a 27% increase of RF production [87]. In the flavinogenic fungus *A. gossypii*, low mRNA levels of the *RIB* genes hindered the overproduction of RF. Accordingly, overexpression of the *RIB* genes resulted in a significant increase in the RF yield [53]. In *E. coli*, an artificial RF operon called EC10 was constructed by re-arranging the genes of the native RF synthesis pathway from *E. coli* (*ribABDEC*), and placing them under the control of the inducible P*trc* promoter. When expressed in wild-type *E. coli* MG1655 from the a high copy number plasmid p20C-EC10, it led to the production of 229 mg/L RF in shake flasks [54]. Overexpression of the *ribB* gene encoding DHPB synthase promoted RF biosynthesis [98]. The EC10 operon was also expressed from a low copy number plasmid to alleviate the metabolic burden of heterologous expression [109]. In *C. ammoniagenes*, a plasmid containing the native RF biosynthetic genes led to 17-fold higher RF accumulation compared to the host strain [88]. In *C. famata*, overexpression of the *RIB1* and *RIB7* genes encoding GTP cyclohydrolase II and RF synthase led to a significant increase of the RF titer [30]. The *RIB1* and *RIB3* genes from *A. gossypii*, which respectively encode GTP-cyclohydrolase II (GCH II) and DHPB synthase were overexpressed in *E. ashbyi* to achieve RF overproduction [89].

Duplication of some or all genes involved in the synthetic pathways of RF is an obvious choice for enhanced RF production. However, the expression of genes responsible for the synthesis of RF can also be improved without increasing their copy number. For instance, mutants resistant to the RF analogue roseoflavin were found to have deregulated transcription of the RF operon due to mutations in the *ribC* gene encoding the bifunctional RFK/FMNAT [52]. The mechanism of the deregulated transcription of the RF operon is thought to be relied to the FMN riboswitch (RFN element) triggered by a decrease of the intracellular FMN concentration [110, 111]. Overexpression of the RF operon was also achieved by replacing the native *rib* promoters with strong constitutive promoters from the SPO1 phage or the P15 promoter to eliminate the feedback inhibition of the natural RF operon [52]. In *E. coli*, downregulating the expression of *ribF* (bifunctional RFK/FMNAT) by changing its RBS effectively increased the production of RF [54].

### Overexpression of the purine biosynthesis pathway

GTP, which is produced by the purine pathway, is a key precursor for the synthesis of RF. Consequently, increased expression of the purine pathway can effectively enhance the production of RF. Overexpression of the purine pathway has been accomplished by mutagenesis and metabolic engineering. The mutagenesis method is suitable for the breeding of *B. subtilis* via resistance selection using structural analogues of purines. Mutants resistant to purine analogues, decoyinine or methionine sulfoxide showed a deregulation of purine synthesis and increased flux from inosine monophosphate (IMP) to guanosine monophosphate (GMP) [52, 112, 113]. Metabolic engineering of the purine pathway includes the overexpression of enzyme-coding genes, as well as the disruption of regulatory genes and competing pathways, especially the pyrimidine pathway.

Improved flux through the purine pathway has been accomplished by various metabolic engineering strategies. Firstly, the overexpression or mutation of key enzymes can be used to increase synthesis of the key purine GTP. In *B. subtilis*, the introduction of an additional copy of the *purF* gene encoding PRPP amidotransferase under the control of the *P43* promoter was able to activate the purine pathway, which led to a 31% increase in the RF titer over the parent strain [91]. Co-overexpression of *purFMNHD* genes improved the titer and yield of RF [91]. A *purF*-VQW mutant strain showed increased PRPP amidotransferase activity and strong resistance to inhibition by purine nucleotides [92]. Increasing the enzyme activity of PRPP synthetase facilitated the production of RF by *A. gossypii* [93]. In this fungus, the reaction catalyzed by the enzyme inosine-5′-monophosphate dehydrogenase (IMPDH) was found to be a rate-limiting step in the guanine nucleotide de novo biosynthetic pathway. Accordingly, overexpression of the IMPDH gene led to a 40% increase of RF production due to an increase of the metabolic flux through the guanine pathway [94]. In *E. coli*, enhanced biosynthesis of GTP was achieved by overexpressing the GTP biosynthesis genes (*ndk* and *gmk*), as well as key genes involved in the purine pathway (*purF* and *prs*) [98].

Secondly, purine synthesis was also increased by derepression of the regulator. In *B. subtilis*, disruption of the *pur* operon repressor *PurR* and the 5′-UTR of the *pur* operon, which contains a guanine-sensing riboswitch, significantly increased the carbon flux through the purine biosynthesis pathway, which further improved the synthesis of RF [92]. In *A. gossypii*, purine biosynthesis is controlled by a Myb-related transcription factor (*AgBAS1*), and its inactivation caused RF overproduction [95]. Furthermore, the purine pathway of *A. gossypii* was modified by constitutive overexpression of the *AgADE4* gene encoding PRPP amidotransferase to abolish adenine-mediated transcriptional repression, as well as site-directed mutagenesis of the *ADE4* gene encoding the PRPP amidotransferase to deregulate the feedback inhibition by purines, leading to a tenfold increase of RF production [96].

Moreover, purine synthesis was also improved by blocking the competing pyrimidine biosynthesis pathway, which led to improved RF production [97]. Optimization of the expression of genes involved in the AMP branch, which competes for purinogenic precursors, also resulted in higher RF production [53].

### Optimization of the central carbon metabolism

While the overexpression of the purine pathway is an efficient strategy for increasing RF production, the carbon flux through the purine pathway itself is limited by the flux of metabolites coming from the pentose phosphate (PP) pathway. Consequently, a further increase of RF production requires enhancing the metabolic flux through the PP pathway. Accordingly, the overexpression of the genes implicated in the PP pathway facilitated the production of RF. For instance, increased expression of the *zwf* gene encoding the glucose-6-phosphate dehydrogenase in *B. subtilis* improved the carbon flux into the PP pathway, which led to a 25% improvement of RF production [100]. Similarly, the co-overexpression of mutant *zwf243* and *gnd361* (encoding 6-phosphogluconate dehydrogenase) from *Corynebacterium glutamicum* (*C. glutamicum*) led to a 39% improvement of RF production [101]. Moreover, overexpression of the *gdh* gene encoding glucose dehydrogenase under the control of the constitutively expressed *P43* promoter was also able to increase the intracellular pool of ribulose 5-phosphate, which increased RF production by 56% [114].

Gluconeogenesis (GNG) produces glucose from certain non-carbohydrate carbon sources, and enhancing it can also stimulate the production of RF. Overexpression of *gapB* (encoding NADPH-dependent glyceraldehyde-3-phosphate dehydrogenase), *fbp* (encoding fructose-1,6-bisphosphatase) and *pckA* (encoding phosphoenolpyruvate carboxykinase) in *B. subtilis* led to the deregulation of gluconeogenesis, which resulted in a 21.9% increase of RF production [102]. The deregulated expression of the gluconeogenetic genes *gapB* and *pckA* was also accomplished by knockout of the genetic repressor CcpN [103].

Disruption of glycolysis (EMP) can improve RF production by enforcing the carbon flux into the PP pathway. In *E. coli*, deletion of the first gene in glycolysis (*pgi* encoding glucose-6-phosphate isomerase) and genes of the Entner–Doudoroff (ED) pathway forcibly redirected the carbon flux into the oxidative PP pathway because it completely diverted the carbon metabolism from the EMP [54]. Similarly, a knockout of the *pfkA* gene encoding 6-phosphofructokinase I resulted in a downregulation of the EMP instead of a complete blockage [109]. This avoided problems of inefficient synthesis of glycine, which is an important precursor in purine synthesis.

### Enhanced synthesis of glycine

The supplementation of the precursor glycine in the medium was helpful for the production of RF by *A. gossypii* [105]. As a precursor of purine biosynthesis, glycine is also a limiting factor for RF production in *A. gossypii*. Overexpression of the *GLY1* gene (encoding threonine aldolase) under the control of the *TEF* promoter and terminator, together with threonine supplementation in the culture medium, led to a tenfold increase of threonine aldolase specific activity and ninefold increase of RF production [104]. This was explained by an enhancement of the intracellular availability of glycine. Disruption of the *SHM2*, gene encoding serine hydroxymethyltransferase, caused a redistribution of carbon fluxes away from serine and toward glycine, which resulted in a significant increase of RF production in *A. gossypii* [105]. Expression of the *AGX1* gene (encoding alanine-glyoxylate aminotransferase) from *Saccharomyces cerevisiae* (*S. cerevisiae*) helped to convert glyoxylate into glycine, which led to 30% increase of RF production in *A. gossypii* [106].

### Other strategies for the optimization of RF-producing microbial cell factories

The optimization of the whole cell chassis for RF production has been implemented in several aspects. Firstly, the optimization of electron transport contributed to the overproduction of RF. Redirection of electron flow to more efficient proton pumping branches within respiratory chains is a generally applicable metabolic engineering strategy for improving product and biomass yields [107]. A knockout of cytochrome bd oxidase (*cydC* deletion) in *B. subtilis* led to a 40% reduction of the rate of maintenance metabolism, which significantly improved the yield of RF and biomass [107]. Redirecting electron flow to a terminal oxidase with high coupling efficiency led to an increased specific growth rate and higher biomass yield, as well as a 30% improvement of RF the biosynthesis ability [115].

Secondly, enhanced export of RF may also be useful to increase the RF yield. Although the transport of RF out of the cell has not been studied in detail, the RF over-producing microorganisms can efficiently passively excrete or actively secrete RF, leading to accumulation in a medium. Heterologous expression of the codon optimized *ribMopt* gene from *Streptomyces davaonensis* (*S. davawensis*), encoding a putative facilitator of RF uptake, may be useful for the promotion of RF production by *B. subtilis* [116].

Moreover, enhanced RF production can also be achieved by engineering an improved robust host. For example, heat shock proteins (HSPs) were expressed in RF overproducing *B. subtilis*, leading to enhanced heat- and osmotic stress tolerance. The stress-tolerant strain showed 23–66% increased RF titers and a quicker fermentation, which was shortened by 24 h [108].

### Systems biology research strategy

As most of the industrially viable RF overproducers were constructed by a combination of classical mutagenesis and genetic engineering, it is necessary to pinpoint the exact genetic characteristics that are responsible for the overproduction of RF. Transposon-tagged mutagenesis, omics techniques and metabolic flux analysis have been applied to reveal the relationship between specific genetic characteristics and the RF overproduction phenotype. In addition, some novel strategies have also been successfully utilized to improve RF production.

The industrial RF producer *B. subtilis* RB50::pRF69 was engineered using a random, transposon-tagged mutagenesis approach to identify novel targets for metabolic engineering [103]. About 10,000 random, transposon-tagged mutants were generated and screened. Then, the transposon insertion sites of both RF overproducing and deficient mutants were analyzed. Subsequently, a novel target, the repressor CcpN, was revealed by reverse engineering.

Transcriptome analysis was also applied to understand the genetic changes in an RF over-producing strain of *B. subtilis* [117]. It was found that the *pur* operon and other PurR-regulated genes were all downregulated in the overproducer. Based on the analysis, the *prs* (encoding PRPP synthetase) and *ywlF* (encoding ribose-5-phosphate isomerase B) were co-overexpressed, which led to a 25% increase of the RF titer.

Another integrated whole-genome and transcriptome sequence analysis of an RF-overproducing *B. subtilis* revealed positive mutations in genes including *ribC* (G199D), *ribD *+ (G + 39A), *purA* (P242L), *ccpN* (A44S) and *yvrH* (R222Q) [118]. Notably, this was the first report that a mutation in *yvrH* (R222Q) can deregulate the purine pathway for improved RF production.

A ^13^C label based metabolic flux analysis (MFA) of an *A. gossypii* strain capable of overproducing RF on vegetable oil was carried out to better understand the underlying metabolic pathways [119]. Unlike the bacterium *B. subtilis*, the fermentation process of the fungus *A. gossypii* exhibited an obvious two-phase profile with an initial growth phase and a subsequent RF production phase. During growth, the TCA cycle was highly active, whereas the flux through gluconeogenesis and the PP pathway was rather low. Yeast extract was the main carbon donor for anabolism, while vegetable oil selectively contributed to the amino acids glutamate, aspartate, and alanine. During the RF biosynthesis phase, the carbon flux through the TCA cycle remained high, and most of the carbon in RF (81 ± 1%) originated from rapeseed oil.

Finally, optimization of the fermentation process and medium composition is vital for achieving an industrially viable level of RF production. This approach has been applied in various RF producers such as *B. subtilis* [120–124], *P. guilliermondii* [125], *Bacillus tequilensis* (*B. tequilensis*) [123], and *Aspergillus terreus* (*A. terreus*) [126, 127]. In *A. gossypii*, a reduction of the growth rate caused by a downshift in the dilution rate during continuous cultivation resulted in a peak of RF overproduction [128].

### Biotechnology of RF by Lactic acid bacteria

Lactic acid bacteria (LAB) is not a traditional overproducer of RF because the RF production of LAB is rather low (no more than 10 mg/L generally). Genetic engineering and chemical analogues screening approaches have been applied for improved RF production of LAB. Detailed introductions can be found elsewhere [1, 14, 15]. The LAB is widely used in the food industry, especially in the dairy industry, which makes it an advantage of RF producing LAB for the food fortification happens in situ.

Recently, researchers have isolated *Lactobacillus* species, which were capable of overproducing RF, from dairy and nondairy sources as well as plant sources [19]. Among the 40 isolates identified as *Lactobacilli*, KTLF1 (*L. fermentum*) and KTLP13 (*L. planatrum*) have been observed to produce an appreciable amount of RF in MRS broth and RF assay (RF-deficient) medium. The RF production of these strains was about 2.1–2.7 mg/L.

In another study, as many as 60 *Lactobacilli* were screened for the ability of RF overproducing via screening of the genes responsible for RF synthesis by a polymerase chain reaction (PCR)-based method [16]. Among the *Lactobacilli* screened, the presence of genes responsible for RF synthesis was strain-specific across different species. The *L. fermentum, L. plantarum, Lactobacillus delbrueckii subsp. bulgaricus,* and *L. mucosae* possessed complete rib structural genes. The other isolates showed incomplete rib structural genes or absence of related genes. The isolates possessing incomplete rib structural genes could not survive in the riboflavin-deficient medium (RAM). On contrary, the isolates KTLF1 (*L. fermentum*) KTLP13 (*L. plantarum*) and KTLF3 (*L. fermentum*) were not only able to grow well on RAM agar but also supported the growth of the RF auxotroph strain. The study also showed that the *Lactobacilli* isolated from human faeces and fermented bamboo shoots possessed maximum RF production. The RF producing *Lactobacilli* isolated was evaluated to be potential probiotic and development of RF bio enriched probiotic food [17].

The soymilk fermented by a RF-producing *L. plantarum* CRL 2130 was able to significantly attenuate trinitrobenzene sulfonic (TNBS)-induced intestinal damages in a murine model [18]. The RF-producing phenotype in LAB represented a potentiality to prevent/treat inflammatory bowel diseases (IBD) [129, 130]. Moreover, the *L. plantarum* CRL 2130 could be useful to prevent mucositis during cancer treatments and would not affect the primary treatment [131].

Incorporation of *Lactococcus lactis* N8 (*L. lactis* N8) and *Saccharomyces boulardii* SAA655 (*S. boulardii* SAA655) led to 40–90% increase of RF and folate in idli batter, a traditional cereal-legume based steamed cake widely consumed in the Indian subcontinent, which indicated the enhanced functional and technological characteristics of idli batter [132]. The screened Andean LAB strains might be useful for the production of cereal-based kefir-like RF-enriched beverages in situ [133]. The quinoa pasta fermented with the LAB *L. plantarum* present increased B2 and B9 levels in mice blood, which indicated that the LAB could be used for preventing nutritional deficiencies [134].

## Whole-cell biocatalysis for FMN and FAD production

Early methods for the production of FAD were based on the whole-cell biocatalytic conversion of its precursors. Different species of bacteria, actinomycetes, molds, and yeasts were screened for their ability to produce FAD from FMN and adenine monophosphate (AMP) [135]. Among the tested microorganisms, bacteria and actinomycetes were able to accumulate detectable amounts of FAD, while yeasts and molds were not. Species belonging to the genera *Sarcina* and *Brevibacterium* produced considerable amounts of FAD, and *Sarcina lutea* (*S. lutea*) was selected especially for FAD production. The maximum concentration of FAD reached 198 mg/L after 144 h of cultivation in the optimized medium containing sucrose and salts, as well as 2% ammonium acetate, 11 mg/ml DCW, 0.1% adenine and 0.1% FMN.

However, the addition of the expensive FMN precursor and the low rate of its conversion to FAD precluded the industrialization of this process. To overcome these disadvantages, a mutant of *S. lutea* deficient in adenosine deaminase was used for the conversion of exogenously supplemented RF to extracellular FAD [136]. The yield of FAD was stimulated by the addition of D-cycloserine due to improved permeability of the cell membrane. The titer of FAD reached 0.7 g/L after 5 days of cultivation in optimal medium with an appropriate amount of thiamine, acetate, and sodium. The yield of FAD isolated by adsorption and ion exchange chromatography was 70%.

In subsequent studies *C. ammoniagenes* was engineered for the production of FMN and FAD by overexpressing its own bifunctional FAD synthetase gene [34]. The maximum FMN titer reached 3.89 mM, representing a 97% molar yield from 4.0 mM RF as substrate after 45 h of cultivation. For the production of FAD, the reaction mixture contained 160 mg/mL of the whole-cell biocatalyst, 23.3 mM FMN (70% purity, net 16.3 mM) or 3.2 mM RF as substrate. With FMN as the precursor, the maximum FAD titer was about 15.3 mM, corresponding to a 94% molar yield. However, RF yielded 1.6 mM FAD, corresponding to only 50% of the molar substrate concentration. The addition of expensive FMN and ATP precursors were the main disadvantages of this method. The cloned FAD synthetase gene of *C. ammoniagenes* could initially not be expressed in *E. coli* because it did not recognize the native promoter of the structural gene [34]. Consequently, the FAD synthetase of *C. ammoniagenes* was expressed in *E. coli* using the tandem tryptophan promoter [33]. The FAD synthetase activity of the recombinant *E. coli* was 2231 times higher than that of wild-type *C. ammoniagenes*. However, its application for the production of FMN and FAD was not reported yet.

A challenging issue immanent in the synthesis of FAD is the concomitant production of FMN, and vice versa. In the whole-cell biocatalytic production of FAD using *C. ammoniagenes,* this problem was efficiently solved by changing the precursors to phosphate polymers with no adenylyl moiety [137]. Among the phosphate compounds tested, only the metaphosphate with no adenylyl moiety could promote the phosphorylation of RF to FMN without inducing the concomitant accumulation of FAD. In the optimized reaction mixture containing 10 mM MgCl_2_, 3 mg/ml metaphosphate, and high concentrations of the enzyme preparation (400 μg/ml), the 40 μM RF added to the reaction mixture was almost completely converted into FMN after 6 h of incubation.

A method for producing flavin nucleotides was patented in 1996 [32]. In the method, a whole or part of a gene, or a gene mutated at the 23^rd^ glycine residue encoding the enzyme which retained RFK and had no or reduced FMNAT activity, was overexpressed in *E. coli*. In an example, a 10 ml reaction mixture produced 18.5 g/L FAD-Na_2_ from 20 g/L FMN and 25 g/L ATP after 24 h, catalyzed by 100 g/L *E. coli* DH5α/pFK5A with the addition of 10 ml xylene and 4 g/L polyoxyethylene-stearylamine. Another example reaction mixture contained (not limited to) 20 g/L *E. col* DH5α/pFK5A, 100 g/L glucose, and 16 g/L RF. In this case, the production of FMN-Na_2_ reached 15.1 g/L with the accumulation of 1.2 g/L FAD-Na_2_. When the cells of *E. coli* DH5α/pFK5A were substituted with the DH5α/pKK12 biocatalyst with the Gly23Ala mutation, the production of FMN-Na_2_ decreased to 10.1 g/L, but the concentration of FAD-Na_2_ also dropped significantly to 0.05 g/L.

## Microbial cell factories for the production of FMN and FAD

### Microbial cell factories for the production of FMN

The flavinogenic yeast *C. famata* was utilized in the industrial synthesis of RF. Recombinant strains of *C. famata* containing the *FMN1* gene (encoding the RFK) driven by the strong *TEF1* promoter exhibited a six- to eightfold increase of RFK activity [138]. Thus, it was reasonable to utilize it industrially, and *C. famata* strains were improved by metabolic engineering for FMN production [36]. The *FMN1* gene (encoding RFK) from *D. hansenii* CBS 767 driven by the strong *TEF1* promoter was introduced into *C. famata* using an integrative vector. The isolated transformants had 3–8 copies of the recombinant *FMN1* gene integrated into the genome. Both the RFK activity and the FMN production increased with the additional copies of the *FMN1* gene. The strains containing 6–8 copies of the *FMN1* gene exhibited a 250-fold increase of RFK and a 40-fold increase of FMN accumulation compared to the wild-type strain. The FMN concentration was 26 mg/L, accounting for 80% of the total excreted flavin content. The integration of the *FMN1* gene was also applied in the *C. famata* strain AF-4, an RF overproducer [139]. The isolated strain possessing 3–4 copies of the *FMN1* gene produced 72 mg/L FMN, accounting for 60% of total flavins. Afterwards, the fermentation of the recombinant strain was optimized using a combination of Plackett–Burman design and central composite design [140]. The maximum production of FMN in the optimized medium reached 231 mg/L after 40 h of fermentation in a 1 L fermenter. However, the FMN titer dropped gradually to about 160 mg/L after 63 h.

A new method called cofactor trapping, which is distinct from traditional metabolic engineering of strains, was developed to produce FMN [141]. This method utilizes an overexpressed flavoprotein to trap FMN. The blue-light photoreceptor protein PpSB2-LOV from *Pseudomonas putida*, which has been modified to exclusively bind FMN [142], was overexpressed in *E. coli* BL21 (DE3). The flavoprotein, as well as all the cellular proteins contained in the crude cell lysate, were denatured using cold perchloric acid to release FMN from the flavoprotein. The yield reached 0.2–0.5 mg FMN/6.5 g DCW, which was 5–10% of the theoretical value. HPLC analysis revealed that the purity of FMN was more than 95%, and it was free from other flavin species. The high purity of the FMN produced this way is a distinct advantage, but the titer was extremely low.

Another method aimed to produce highly pure FMN by compartmentalizing the final FMN biosynthesis step into the periplasm [143]. The synthesis of FMN outside the cytoplasm helped to eliminate the undesirable accumulation of RF and FAD in the spent medium. A two-plasmid system was used in *E. coli* for the respective overexpression of the cytoplasmic proteins, as well as the membrane and periplasmic enzymes. The *RibADEH* genes responsible for RF synthesis and the *RibC* gene encoding a bifunctional RFK/FADS from *B. subtilis* were integrated successively into the vector for the overproduction of RF and its further conversion into FAD in the cytoplasm. Another vector bearing the *SO0702* gene encoding the inner membrane FAD exporter and the *UshA* gene encoding the periplasmic 5′-nucleotidase from *Shewanella oneidensis* (*S. oneidensis*) MR-1 was used for the transport of FAD into the periplasm and its hydrolysis to FMN. The final engineered strain produced 70.8 mg/L FMN, accounting for 92.4% of the total excreted flavins.

Engineered *E. coli* strain was capable of overproducing RF [109]. The *ribF* gene encoding the bifunctional RFK/FADS from *E. coli* was overexpressed in the previously engineered RF overproducing strain [38]. The resulting strain was able to overproduce FMN and FAD simultaneously. Next, the *ribF* gene was modified by error-prone PCR. The strains harboring the mutant *ribF* gene were able to overproduce FMN with no FAD or trace amounts of FAD in the culture supernatants. The final FMN concentration reached 1017 mg/L in a 5 L fermenter in fed-batch fermentation mode.

### Microbial cell factories for the production of FAD

The yeast *C. famata* was also engineered for FAD production by overexpressing the *FAD1* gene encoding the monofunctional FAD synthetase [37]. In analogy to the FMN overproducing strain, one or multiple copies of the *FAD1* gene encoding the monofunctional FADS from *D. hansenii* CBS 767 or the *FAD1* gene together with the *FMN1* gene [36] encoding RFK were integrated into the genome of *C. famata*. Expression of the *FAD1* gene led to 7–15 fold increase of FAD activity. The isolated strain containing chromosomal copies of both the *FMN1* and *FAD1* genes (*C. famata* T-FD-FM 27) had the highest FAD titer of 63 mg/L, with 101–154 mg/L FMN accumulation after 48 h of growth. Next, the production of FAD was further improved by optimizing the culture conditions. The titer of FAD reached 387 mg/L at its peak in the optimized medium in shake flasks fermentation, but later dropped sharply to about 120 mg/L. The maximum of FAD titer was 451 mg/L after 40 h batch cultivation in a 1.2 L fermenter with optimized medium. The sudden drop of FAD concentration in this cultivation process was accompanied by a steep rise of RF production.

As an important cofactor, FAD plays a key role in many enzymatic reactions. An increased intracellular FAD concentration in *E. coli* improved the production of phenylpyruvic acid (PPA) [144]. Genes related to the synthesis of FAD, including *ribADBHCF*, were overexpressed individually to investigate their effects on the intracellular FAD concentration, and the results suggested that reactions catalyzed by *ribC* (encoding RF synthase), *ribF* (encoding the bifunctional RFK/FADS) and *ribH* (encoding lumazine synthase) were the main rate-limiting steps for FAD biosynthesis. The simultaneous overexpression of *ribC, ribF* and *ribH* led to an increase of the intracellular FAD concentration and improved PPA production. The resulting FAD concentration (1.16 mM) and PPA production (23.2 g/L) were 2.2 and 2.3 times higher than the corresponding values of the control. Moreover, the strengthening of the FADH_2_/FAD cofactor regeneration rate resulted in a further improvement of PPA production to 31.4 g/L.

Engineering of *E. coli* for the overproduction of RF for FAD production has also been accomplished. In order to obtain strains possessing a high FAD production capacity with a high conversion ratio, modular engineering of the flavin pathway was carried out [38]. The RF operon responsible for the synthesis of RF and the *ribF* gene encoding the bifunctional RFK/FADS were divided into two separate modules, which were then expressed at different levels and ratios. Both the ratio and the expression strength of the two modules had a significant effect on flavin production and the conversion ratio of RF (cr-RF). The best strain respectively produced 324 and 171 mg/L of FAD and FMN in shake flask fermentation with a cr-RF of 0.78. The final titers of FAD and FMN in a 5 L bioreactor respectively reached 1899 and 872 mg/L, with a cr-RF of 0.73.

## Conclusions

The industrial biosynthesis of RF is a great success of microbial fermentation technology. Despite the fact that industrial RF synthesis is exclusively achieved by fermentation, most of the strains used in the industrial field were isolated by mutagenesis, which made their genomic background ambiguous. Researchers have tried to identify the genes and genetic elements responsible for the RF overproduction phenotype through different techniques, including transcriptome analysis [117] and screening of transposon-tagged mutants [103], as well as the more recently reported integrated whole-genome and transcriptome sequence analysis [118] and metabolic flux analysis [145, 146]. Novel “omics” techniques such as metabolomics may prove useful in revealing further details of the flavin overproduction mechanism. Recently, the European Food Safety Authority (EFSA) Panel on Additives and Products or Substances used in Animal Feed (FEEDAP) delivered a scientific opinion on the RF produced using *Bacillus subtilis* KCCM-10445 as a feed additive for all animal species. The panel concluded that 80% pure RF poses a risk for the spread of viable cells and DNA of genetically modified strains harboring antibiotic resistance genes [31]. However, FEEDAP could not draw a final conclusion on the risk posed for the user by inhalation of RF [147, 148] or monosodium riboflavin 5′-phosphate [148] produced using *A. gossypii*. The production strain *B. subtilis* KCCM-10445 carries four antimicrobial resistance genes, three of which were introduced by genetic modifications. Thus, it is necessary to develop RF overproducing strains by metabolic engineering or genome editing with a clear genetic background, and either with as few as possible, or without antimicrobial resistance genes.

The industrial-scale synthesis of FMN proceeds via the phosphorylation of RF using nonspecific phosphorylation reagents, which is accompanied by the formation of isomeric FMN byproducts [21]. Consequently, commercial-grade FMN and FAD are much more expensive than RF. Previous work has proven that overexpression of RFK/FMNAT in RF-overproducing strains can lead to the overproduction of FMN and FAD [36–38]. However, the available strains for FMN and FAD production still have significant deficiencies, since they generally produce a mixture of RF, FMN and/or FAD, with a low titer of the latter. These issues remain to be resolved to develop strains that can produce FMN and/or FAD with high yield and purity.

## Data Availability

Not applicable.

## (Pathway)

PP pathway: The pentose phosphate pathway
TCA: Tricarboxylic acid cycle
EMP: Embden–Meyerhof pathway/glycolysis
ED: Entner–Doudoroff pathway

## (Metabolites)

G6P: Glucose-6-phosphate
GAP: Glyceraldehyde 3-phosphate
3GP: Glycerate 3-phosphate
PYR: Pyruvate

## OAA, oxaloacetate

6-Pgdl: 6-Phospho-d-glucono-1, 5-lactone
gluconate-6P: 6-Phospho-d-gluconate
PRPP: Phosphoribosylpyrophosphate
Ru5P: Ribulose 5-phosphate
GTP: Guanosine 5′-triphosphate
IMP: Inosine monophosphate
IMPDH: Inosine-5′-monophosphate dehydrogenase
GMP: Guanosine monophosphate
AMP: Adenosine monophosphate
ATP: Adenosine triphosphate
Gly: Glycine
GTP: Guanosine 5′-triphosphate
DHPB: L-3,4-Dihydroxybutan-2-one 4-phosphate
DARPP: 2,5-Diamino-6-(1-d-ribosylamino)pyrimidin-4(3H)-one 5′-phosphate
AFAPP: 2-Amino-5-formylamino-6-(5-phospho-d-ribosylamino)pyrimidin-4(3H)-one
DArPP: 2,5-Diamino-6-(1-d-ribitylamino)pyrimidin-4(3H)-one 5′-phosphate
ARPP: 5-Amino-6-(ribosylamino)-2,4-(1H,3H)-pyrimidinedione 5′-phosphate
ArPP: 5-Amino-2,6-dioxy-4-(5′-phospho-d-ribitylamino)pyrimidine
ArP: 5-Amino-6-(1-d-ribitylamino)uracil
DrL: 6,7-Dimethyl-8-(D-ribityl)lumazine
RF: Riboflavin
FMN: Flavin mononucleotide
FAD: Flavin adenine dinucleotide
RoF^r^: Roseoflavin
Az^r^: 8-Azaguanine resistance
Dc^r^: Decoyinine resistance
AMP: Adenosine monophosphate:
ATP: Adenosine triphosphate
RFK: Riboflavin kinase
FMNAT: FMN adenylyltransferase
FADS: FAD synthetase

## (Others)

cr-RF: The conversion ratio of riboflavin
EFSA: European Food Safety Authority
FEEDAP: Products or Substances used in Animal Feed
